# Global Diversity of Desert Hypolithic Cyanobacteria

**DOI:** 10.3389/fmicb.2017.00867

**Published:** 2017-05-16

**Authors:** Donnabella C. Lacap-Bugler, Kevin K. Lee, Stephen Archer, Len N. Gillman, Maggie C.Y. Lau, Sebastian Leuzinger, Charles K. Lee, Teruya Maki, Christopher P. McKay, John K. Perrott, Asunción de los Rios-Murillo, Kimberley A. Warren-Rhodes, David W. Hopkins, Stephen B. Pointing

**Affiliations:** ^1^Institute for Applied Ecology New Zealand, School of Science, Auckland University of TechnologyAuckland, New Zealand; ^2^Department of Geosciences, Princeton University, PrincetonNJ, USA; ^3^International Centre for Terrestrial Antarctic Research, School of Science, University of WaikatoHamilton, New Zealand; ^4^Institute of Nature and Environmental Technology, Kanazawa UniversityKanazawa, Japan; ^5^National Aeronautics and Space Administration Ames Research Center, Moffett FieldCA, USA; ^6^Departamento de Biogeoquímica y Ecología Microbiana, Museo Nacional de Ciencias NaturalesMadrid, Spain; ^7^The Royal Agricultural UniversityCirencester, UK

**Keywords:** biogeography, cyanobacteria, desert, dryland, hypolith

## Abstract

Global patterns in diversity were estimated for cyanobacteria-dominated hypolithic communities that colonize ventral surfaces of quartz stones and are common in desert environments. A total of 64 hypolithic communities were recovered from deserts on every continent plus a tropical moisture sufficient location. Community diversity was estimated using a combined t-RFLP fingerprinting and high throughput sequencing approach. The t-RFLP analysis revealed desert communities were different from the single non-desert location. A striking pattern also emerged where Antarctic desert communities were clearly distinct from all other deserts. Some overlap in community similarity occurred for hot, cold and tundra deserts. A further observation was that the producer-consumer ratio displayed a significant negative correlation with growing season, such that shorter growing seasons supported communities with greater abundance of producers, and this pattern was independent of macroclimate. High-throughput sequencing of 16S rRNA and *nif*H genes from four representative samples validated the t-RFLP study and revealed patterns of taxonomic and putative diazotrophic diversity for desert communities from the Taklimakan Desert, Tibetan Plateau, Canadian Arctic and Antarctic. All communities were dominated by cyanobacteria and among these 21 taxa were potentially endemic to any given desert location. Some others occurred in all but the most extreme hot and polar deserts suggesting they were relatively less well adapted to environmental stress. The t-RFLP and sequencing data revealed the two most abundant cyanobacterial taxa were *Phormidium* in Antarctic and Tibetan deserts and *Chroococcidiopsis* in hot and cold deserts. The Arctic tundra displayed a more heterogenous cyanobacterial assemblage and this was attributed to the maritime-influenced sampling location. The most abundant heterotrophic taxa were ubiquitous among samples and belonged to the Acidobacteria, Actinobacteria, Bacteroidetes, and Proteobacteria. Sequencing using nitrogenase gene-specific primers revealed all putative diazotrophs were Proteobacteria of the orders Burkholderiales, Rhizobiales, and Rhodospirillales. We envisage cyanobacterial carbon input to the system is accompanied by nitrogen fixation largely from non-cyanobacterial taxa. Overall the results indicate desert hypoliths worldwide are dominated by cyanobacteria and that growing season is a useful predictor of their abundance. Differences in cyanobacterial taxa encountered may reflect their adaptation to different moisture availability regimes in polar and non-polar deserts.

## Introduction

Terrestrial ecosystems that experience prolonged moisture deficit are known as deserts or drylands and they comprise the largest terrestrial biome (Laity, 2008). They are categorized in terms of temperature and aridity such that hot, cold, polar tundra and polar frost deserts are differentiated (Peel and Finlayson, 2007). Deserts are further defined as tropical sub-humid, semi-arid, arid or hyper-arid depending on the level of moisture deficit in the system (UNEP, 1992). As aridity increases vascular plants and metazoans decrease in abundance and the importance of microbial communities in ecological processes increases (Pointing and Belnap, 2012; Pointing et al., 2015). A major focus of research has been the hypolithic microbial communities that develop as biofilms on the ventral surfaces of quartz stones (Chan et al., 2012; Pointing, 2016), since this substrate is commonly encountered as ‘desert pavement’ in extreme deserts worldwide (Laity, 2008; Thomas, 2011).

A number of studies have reported biodiversity in hypolithic communities using microscopy and environmental sequencing approaches, and there is consensus from individual studies that they are dominated by cyanobacteria with a relatively cosmopolitan and less abundant heterotrophic assemblage in Africa (Weber et al., 2013), Antarctica (Pointing et al., 2009), Arctic (Cockell and Stokes, 2004), Australia (Tracy et al., 2010), China (Warren-Rhodes et al., 2007), North America (Schlesinger et al., 2003), and South America (Warren-Rhodes et al., 2006). The hypolithic community is clearly distinct from those in other desert niches such as soil, cryptoendolithic communities that colonize weathered porous rocks and chasmoendoliths that colonize cracks and fissures in rock (Chan et al., 2012; Wierzchos et al., 2012).

Major advances in resolving hypolithic microbial biodiversity have been reviewed comprehensively in recent publications (Chan et al., 2012; Pointing, 2016). The different approaches in most studies, however, have made comparison difficult, with only two attempting a global comparative analysis. An ecological modeling study of hypoliths worldwide based upon t-RFLP fingerprinting revealed that photoautotrophic and heterotrophic assemblages within the community developed under different stochastic and deterministic influences (Caruso et al., 2011). A multi-locus phylogenetic study of the desert cyanobacterial genus *Chroococcidiopsis* identified that they displayed a high degree of endemism and could be divided phylogenetically into distinct hot and cold adapted clades (Bahl et al., 2011). Both these studies indicated that cyanobacteria were the most abundant taxa in hypolithic communities, and it has been proposed that they are the main biotic drivers of community assembly and function (Valverde et al., 2015). Meta-analyses of previous studies have suggested that cyanobacterial taxa may display marked variation between hot, cold and polar deserts (Chan et al., 2012; Pointing, 2016), however, this remains unproven in direct comparative studies and the impact on overall community structure is unresolved. Resolving this issue has major implications for understanding productivity and diazotrophy in this oligotrophic system (Pointing et al., 2015), since hypolithic cyanobacteria are the only photoautotrophic bacterial phylum and have been assumed to also fulfill a major ecological role as nitrogen fixers (Cowan et al., 2011b).

Here we hypothesized that hypolithic cyanobacteria display a detectable difference in diversity due to location and/or climate, and that this has an impact on overall community assembly and putative community functionality. We used a global dataset of hypolithic communities from 12 locations spanning major deserts on every continent, and combined t-RFLP fingerprinting and high throughput sequencing of 16S rRNA and *nifH* genes to reveal patterns of taxonomic and putative diazotrophic diversity for hot, cold, tundra and polar desert communities.

## Materials and Methods

### Sample Recovery

Hypolithic communities were recovered as previously described (Pointing et al., 2009) from quartz substrate in desert pavement at locations on every continent, plus a non-desert control site (**Supplementary Table S1**). At each sampling location a minimum of three colonized stones were collected (**Supplementary Table S1**). The samples were obtained opportunistically in a crowd-sourced manner and so sample numbers for each desert region varied. Briefly, colonized quartz stones were retrieved by hand (using isopropyl alcohol surface sterilized latex gloves) and loose soil particles gently removed with a sterile (autoclaved) paintbrush. Samples were then stored in sterile Whirlpak bags (Nasco) at -20°C in the field and in transit, and subsequently stored frozen at -80°C in the laboratory until processed. Some subsequently failed to yield quality DNA and the final sample number analyzed was 64 hypoliths.

### Climate Data

Precipitation and temperature data were obtained from the freely available 10′ gridded global data sets provided by the Climate Research Unit (CRU) of the University of East Anglia (New et al., 2002). Mean yearly precipitation and temperatures were calculated for the given sites, with an accuracy of ±18 km. The number of days per year when temperature, moisture and light allowed photosynthesis to occur was expressed as the growing season (Paulsen et al., 2014). Climate data is summarized in **Supplementary Table S1**.

### t-RFLP Community Fingerprinting

Hypolithic biofilms were scraped from a 2cm^2^ area of colonized quartz stones using a sterile scalpel and forceps. Environmental DNA recovery was achieved separately for each sample by lysis in CTAB with lysozyme and RNAse, followed by phenol:chloroform extraction at 60°C (Pointing et al., 2009). Recovered DNA was quantified using Nanodrop (Thermo-scientific) and template for all samples normalized at 100 ng/DNA per reaction. The PCR reaction comprised a 25 μl PCR mixture containing 0.1–2 μl of DNA template, 0.5 μM of each primer, 2.5 units of high fidelity *Taq* polymerase (Takara, Beijing, China) ^1^, 1x PCR buffer provided by the manufacturer, 200 μM of each dNTP, and ddH_2_O. Amplification of 16S rRNA genes was achieved using primer pair 341F-CCTACGGGAGGCAGCAG and 907R-CCGTCAATTCMTTTGAGTTT (Muyzer et al., 1993). PCR reactions for t-RFLP analysis were carried out using a FAM-labeled forward primer as previously described (Pointing et al., 2009). The PCR reaction involved an initial denaturation time of 5min; 30 cycles at 95°C for 1 min, 55°C for 1 min, 72°C for 1 min, and a final extension at 72°C for 10 min. Positive and negative controls were run for every PCR. Gel-purified amplicons were digested using three restriction enzymes HaeIII, HinfI, MspI (ThermoFisher, Hong Kong, China) ^2^ and the most informative selected for further analysis (MspI). Fragment analysis was achieved by capillary electrophoresis (Applied Biosystems 3730 Genetic Analyzer), using a GeneScan ROX-labeled GS500 internal size standard from the manufacturer. The t-RFLP patterns and quality were analyzed using the freeware PeakScanner^TM^ (version 1.0; Applied Biosystems)^3^ and a data matrix comprising fragment size and abundance was generated. The software Perl and R (R Core Team, 2016) were then used to identify true peaks from artifacts among the terminal restriction fragment sequences and bin fragments of similar size as previously described (Abdo et al., 2006). Peaks within three standard deviations of the baseline noise signal were excluded. The relative abundance of a terminal restriction fragment within a given t-RFLP pattern was generated as a ratio of the respective peak area to total area of all peaks. A virtual digest using MspI was carried out on sequences from our extensive curated 16S rRNA library for Antarctic bacteria (Pointing et al., 2009) and this allowed assignment of phylogenetic identity to 82% of t-RFLP peaks. Those peaks within 1 bp of another were regarded as representing the same taxon.

A non-metric multidimensional scaling plot (NMDS) of Bray–Curtis similarities were generated from t-RFLP defined bacterial communities and groupings of communities in these ordinations were made with a 40% dissimilarity cutoff, using PRIMER 6 (Clarke, 1993). The t-RFLP data were fitted to growing season variables using a simple linear regression model performed using R (R Core Team, 2016). One hot desert outlier sample was removed from this analysis since the value exceeded the mean for the location and overall dataset by an order of magnitude.

### Pyrosequencing and Phylogenetic Assignment

Samples for each statistically supported desert grouping from Bray–Curtis analysis (above) were selected based upon greatest number of shared OTUs from the t-RFLP analysis. These were further interrogated via barcoded pyrosequencing using the Roche GS Junior System (454 Life Sciences Corp., Branford, CT, USA). This resulted in high throughput sequencing of samples as follows: Taklimakan Desert, China; Tibetan Plateau, China; Devon Island, Canadian Arctic; McMurdo Dry Valleys, Antarctica. Amplification of 16S rRNA genes was achieved using primer pair 341F and 907R (Muyzer et al., 1993) with PCR conditions as described above. For each amplicon library purification was carried out with Agencourt AMPure XP Bead (Beckman Coulter, CA, USA) ^4^ according to manufacturer’s instructions. The libraries were quantified with Quant-iT PicoGreen dsDNA Assay Kit (Invitrogen Life Technologies, NY, USA)^2^ using FLUOstar OPTIMA F fluorometer (BMG Labtech GmbH, Offenburg, Germany) ^5^ and library quality was assessed with the FlashGel System (Lonza Group Ltd., Basel, Switzerland). Emulsion-PCR was carried out with GS Junior Titanium emPCR Kit (Lib-L, 454 Life Sciences Corp., CT, USA)^6^ according to the emPCR Amplification Method Manual – Lib-L, Single-Prep. The sequencing reaction was carried out with the GS Junior Titanium Sequencing Kit and GS Junior Titanium PicoTiterPlate Kit (454 Life Sciences Corp.) according to the manufacturer’s instructions. The sequencing run was conducted in 200 cycles. The *nifH* sequence libraries were generated using the primer set, *nif*H forward (5′ TGY GAY CCN AAR GCN GA3′) and *nif*H reverse (5′ADN GCC ATC ATY TCN CC3′) with barcodes in the forward primer. This followed the same methodology as the 16S rRNA pyrosequencing described above. All PCR reactions included positive and negative controls for 16S rRNA and *nif*H genes as appropriate.

Pyrosequencing reads were sorted according to barcoding prior to analysis and processing using the software package MOTHUR (Schloss and Handelsman, 2005). De-noising was carried out with sequences removed from analysis if they met any of the following criteria: the length was shorter than 300 bp; with an average quality score less than 25; contained ambiguous characters or more than 6 homopolymers; did not contain the primer sequence or barcode. In order to remove sequences that were probably due to pyrosequencing errors, sequences were pre-clustered using a pseudo-single linkage algorithm as implemented in MOTHUR. Chimera check was performed using UCHIME with the *de novo* mechanism (Edgar, 2010). Hierarchical clustering was performed with the remaining sequences to form clumps that were small enough to align using USEARCH (Edgar, 2010). A master set was created using the longest sequence from each clump. Sequences in the clumps and master set were aligned using MUSCLE (Edgar, 2004). The aligned sequences were merged into a final alignment with the master set as a guide. Alignment columns containing more than 90% gaps were trimmed using trimAL (Capella-Gutiérrez et al., 2009). Alpha diversity was assessed by constructing the rarefaction curves defined at 97% sequence similarity cutoff for operational taxonomic units (OTUs). Taxonomic classification of 16S rRNA gene sequences was made using the Ribosomal Database Project Classifier (Wang et al., 2007).

For cyanobacterial phylogeographic inference, reads from the four samples were pooled, and the 30 most relatively abundant cyanobacterial OTUs were chosen for further analysis. These 30 representative 16S rRNA gene OTU sequences were aligned with other known cyanobacterial phylotype sequences (Bahl et al., 2011) via MUSCLE (Edgar, 2004). A maximum likelihood phylogenetic tree was inferred using RAxML (Stamatakis, 2014) with the GTRGAMMA model. A bootstrap analysis with 100 replicates was conducted and the result was used for generating bipartition support value on the best scoring tree. Sequence data have been deposited in NCBI’s sequence read archive under accession number PRJEB15586, ERS1374529- 32 for 16S rRNA gene data and ERS1374533-35 for *nifH* gene data.

## Results

**FIGURE 1:**
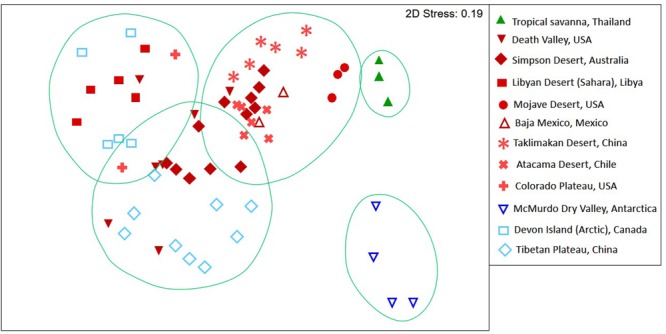
**Non-metric multidimensional scaling plot of Bray–Curtis similarities for t-RFLP defined bacterial communities recovered from hypoliths in major deserts worldwide.** Circles indicate clusters of communities at a 40% dissimilarity threshold. Colors denote macroclimate (Peel and Finlayson, 2007): Polar frost (EF) = dark blue, Polar Tundra (ET) = light blue, Cold desert (BWk) = light red, Hot desert (BWh) = dark red, Tropical savanna (Af) = green.

Patterns in 16S rRNA gene defined diversity for 64 discreet hypolith communities (**Supplementary Table S1**) revealed five distinct groupings at a 40% Bray–Curtis dissimilarity threshold (**Figure 1**). The tropical aquatic hypolithic communities were distinct from all desert samples. The Antarctic hypolithic communities were most distinct from all other communities worldwide. The three remaining desert groupings comprised a largely hot/cold deserts cluster, Tibetan Plateau cluster and Arctic/Sahara cluster. Some overlap between these three clusters occurred, for example hypolithic communities from Australia and California shared similarity across all three clusters, and Arctic and Saharan communities occurred in the same similarity cluster.

**FIGURE 2:**
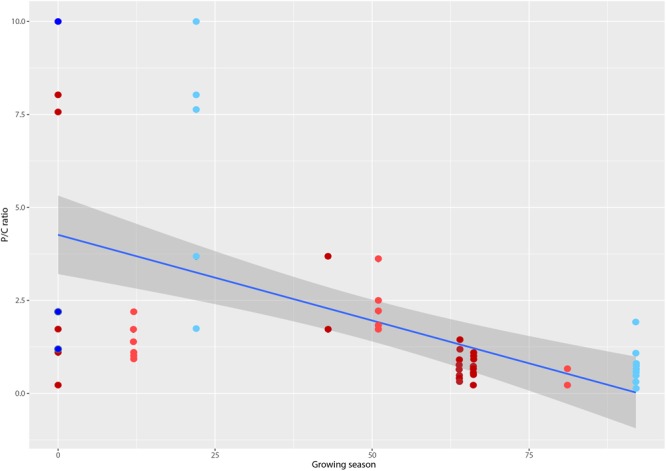
**Plot of producer/consumer ratio (P/C) for hypolithic communities versus growing season, a metric that defines the number of days per year when photosynthesis is possible (d) (Paulsen et al., 2014).** Colors denote macroclimate according (Peel and Finlayson, 2007): Polar frost (EF) = dark blue, Polar Tundra (ET) = light blue, Cold desert (BWk) = light red, Hot desert (BWh) = dark red. Locations from left to right are: McMurdo Dry Valleys, Antarctica; Libyan Desert (Sahara), Libya; Taklimakan Desert, China; Devon Island (Arctic), Canada; Baja Mexico, Mexico; Atacama Desert, Chile; Simpson Desert, Australia; Mojave Desert, USA; Death Valley, USA; Colorado Plateau, USA; Tibetan Plateau, China. Line shows linear regression fit (*R*^2^ = 0.2908, *p* < 0.0001) and shaded area denotes 95% confidence limits.

Resolution of t-RFLP sequence fragments with an extensive hypolithic sequence library (Pointing et al., 2009) allowed identification to at least phylum but in many cases to genus level for 73 of the 89 OTUs. Assignment of phylogenetic identity and binning t-RFLP fragments into autotrophs (Cyanobacteria and chemoautotrophs) and heterotrophs (all other bacteria) allowed calculation of the producer/consumer ratio (P/C). This revealed that P/C varied significantly between lower values in the Colorado Desert and Tibetan Plateau where growing season was highest, to higher P/C values in the Antarctic and Sahara deserts with the lowest growing seasons (**Figure 2**). The most extreme deserts therefore likely supported communities with significantly greater photoautotrophic biomass as a relative percentage of the community than less extreme deserts with longer growing seasons. We identified a weak but significant inverse relationship between P/C ratio and mean annual precipitation (MAP; *R*^2^ = -0.28564, *p* = 0.0102), minimum winter temperature (*R*^2^= -0.28420, *p* = 0.0106) and maximum summer temperature (*R*^2^= -0.28527, *p* = 0.0103). A slightly more significant correlation was observed for growing season (*R*^2^ = 0.2908, *p* < 0.0001), a term extrapolated from combined macroclimate variables that describes the number of days per year when photosynthesis is possible.

**FIGURE 3:**
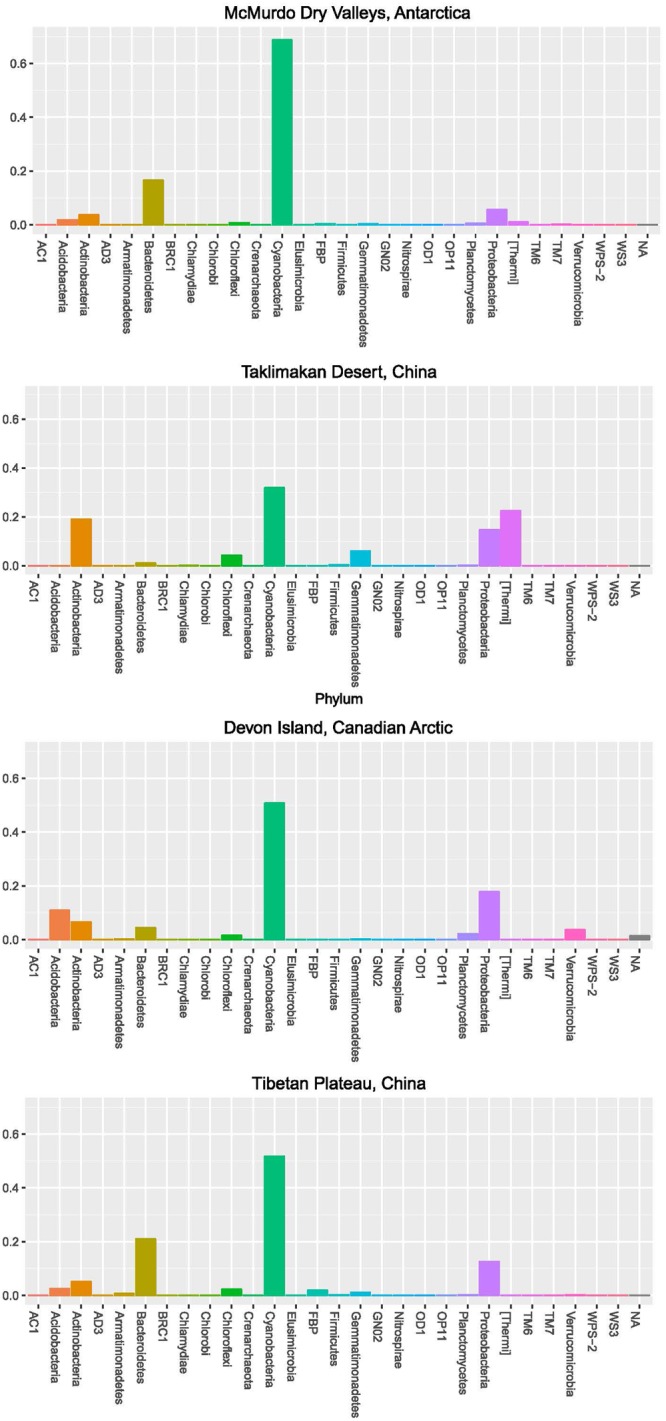
**Community composition for desert hypolithic communities based upon pyrosequencing of the 16S rRNA gene.** Values indicate relative abundance for each phylum. Full identification of all OTUs is listed in **Supplementary Table S2**.

**FIGURE 4:**
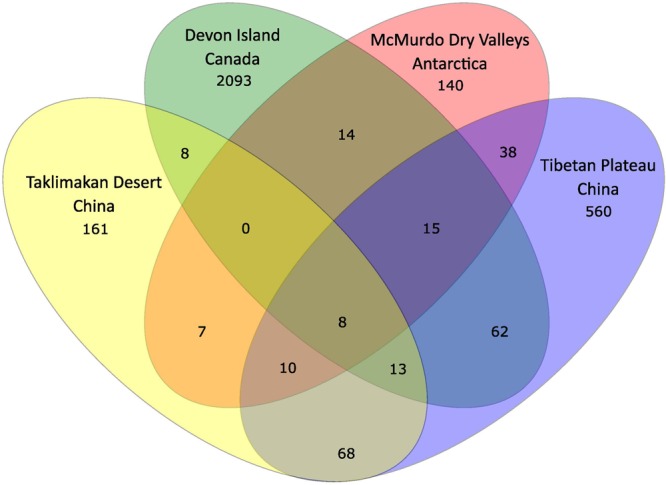
**Venn diagram illustrating distribution of OTUs among the four locations.** Taxonomic classification of 16S rRNA gene sequences was made using the Ribosomal Database Project Classifier (Wang et al., 2007). A full list of all OTUs is given in **Supplementary Table S2**.

In order to further understand the hypolithic biodiversity, we selected the most representative samples from each of the four statistically supported desert hypolith community clusters (based upon greatest number of shared OTUs) and subjected them to high throughput sequencing of the V3–V4 region of the 16S rRNA gene. Among the four samples, a total of 237,653 merged and filtered paired-end reads were generated for this study, with an average length of 344 bp (*SD* = 42.25). The sequencing confirmed and validated the general trend in diversity and P/C observed in the t-RFLP study in that the Taklimakan Desert community was dominated by heterotrophic taxa with approximately 35% cyanobacteria, whilst in Tibetan and Arctic tundra deserts this figure rose to approximately 50%, and in the Antarctic sample cyanobacteria comprised over 70% of taxa (**Figure 3** and **Supplementary Table S2**). The dominant heterotrophic phyla in all samples were Acidobacteria, Actinobacteria, Bacteroidetes, Proteobacteria and Thermi, although the most abundant heterotrophic phylum varied between each sample (**Figure 3**). All four locations displayed a large number of unique OTUs with only eight OTUs shared across all locations and these were largely heterotrophic taxa (**Figure 4** and **Supplementary Table S2**). The geographically most proximal Taklimakan Desert and Tibetan Plateau samples displayed greatest shared similarity, closely followed by the Tibetan and Arctic samples (**Figure 4**). One sample (Arctic) had significantly more OTUs than all other samples but these were generally low abundance taxa comprising less than 1% of the overall community.

**FIGURE 5:**
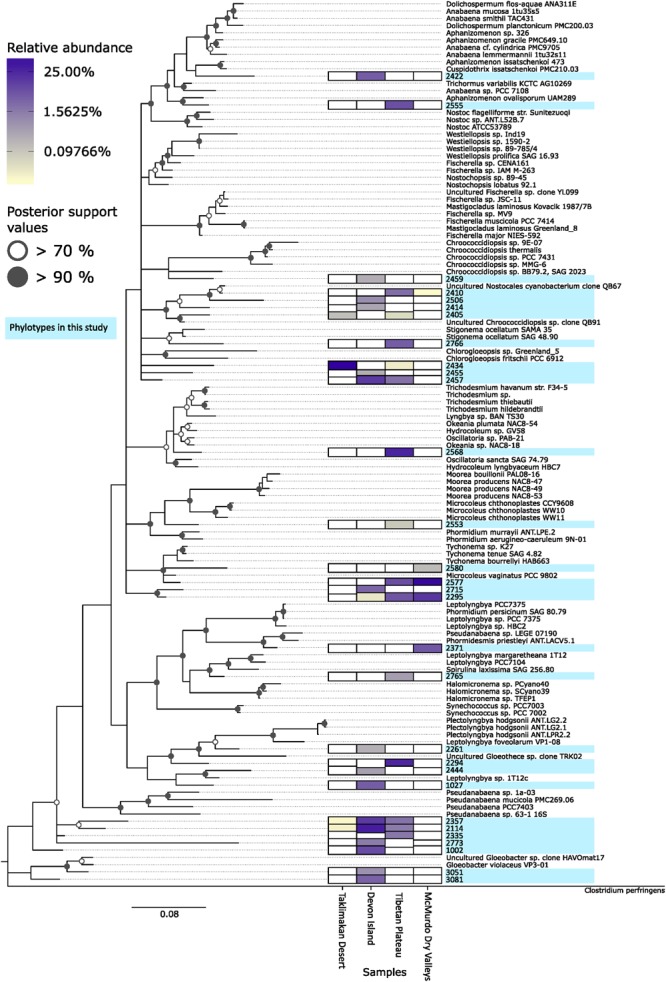
**Phylogenetic tree of desert hypolithic cyanobacteria based upon pyrosequencing data.** Heatmap shows relative abundance of the 30 most abundant cyanobacterial phylotypes in this study for each location. The phylogenetic tree was generated via RAxML (Stamatakis, 2014) with the GTRGAMMA model. A bootstrap analysis with 100 replicates was conducted and the result was used for generating bipartition support value on the best scoring tree. Bifurcation nodes with >70% bootstrap support values were annotated with an open circle, nodes with >90% bootstrap support values were annotated with a filled circle.

The sequence data indicated cyanobacterial taxa displayed a pronounced distribution pattern between the four desert samples (**Figure 5**). There were 21 taxa endemic to any given desert, whilst others occurred in all but the most extreme Taklimakan and Antarctic deserts. For example, OTU 2405 and 2434 were found only in geographically non-Polar deserts, OTUs 2114 and 2357 occurred in all but the extreme Antarctic desert, whilst OTU 2295 occurred in all locations except the Taklimakan Desert. A phylogenetic analysis of cyanobacterial OTUs (**Figure 5**) revealed that potentially endemic taxa spanned several cyanobacterial families. There was also a clear difference in the ratio of the two most abundant genera, *Chroococcidiopsis* and *Phormidium* (**Table 1**). In the Taklimakan desert virtually all cyanobacterial OTUs were *Chroococcidiopsis*, whilst in Antarctic samples they were almost exclusively *Phormidium* sp. Moreover the Antarctic desert was the only hypolithic community that did not support any *Chroococcidiopsis* OTUs.

**Table 1:** Summary of 16S rRNA sequence data.

Putative ecotype		Relative abundance (%)			
		McMurdo Dry Valleys Antarctica	Taklimakan Desert China	Devon Island Canadian Arctic	Tibetan Plateau China
	Growing season (d)	∼0	12	22	92
Producers		68.7	31.9	46.0	51.8
	*Chroococcidiopsis* sp.	0.0	31.9	13.7	4.6
	*Phormidium* sp.	67.8	0.02	22.7	40.7
Consumers		31.3	68.1	54.0	48.2
Diazotrophs		5.3	14.6	13.8	18.4

*Producers include all OTUs indicating cyanobacteria, relative abundance of the *Chroococcidiopsis* and *Phormidium* genera are also given. Consumers include all heterotrophic bacterial taxa, diazotrophs include all taxa belonging to known nitrogen fixing genera. For a complete description of all OTUs please refer to **Supplementary Table S2**.*

This pronounced difference in cyanobacterial distribution led us to ask whether aside from cyanobacterial photoautotrophy if nitrogen fixation, a function also traditionally associated with cyanobacteria in hypoliths and other communities, may be impacted by the biogeography of cyanobacterial assemblages. We screened the 16S rRNA sequences for taxa known to be diazotrophic, and revealed that Taklimakan, Tibetan and Arctic desert supported 14–18% putative nitrogen fixing taxa whilst in the Antarctic sample this dropped dramatically to approximately 5% (**Table 1** and **Supplementary Table S2**). A second high throughput sequencing experiment targeting the *nifH* gene for nitrogenase was therefore conducted, although the number of filtered reads was low compared to the 16S rRNA study. Surprisingly no cyanobacterial *nifH* was recovered, instead all nitrogenase sequences affiliated with the Proteobacteria (**Table 2** and **Supplementary Table S3**). This is despite recovery of 16S rRNA phylotypes indicating the diazotrophic family Nostocales occurred at low abundance in all locations except the hot desert.

**Table 2:** Summary of *nifH* sequence data.

OTU No.	Taklimakan Desert China	Devon Island Canada	Tibetan Plateau China	McMurdo Dry Valley Antarctica	Total	Order
1	0	0	23	0	23	Rhizobiales
2	0	0	12	0	12	Burkholderiales
3	0	0	7	0	7	Rhodospirillales
4	6	0	0	0	6	Rhodospirillales
5	0	0	3	0	3	Unknown
6	2	0	0	1	3	Rhizobiales
7	0	0	0	3	3	Burkholderiales
8	2	0	0	0	2	Rhizobiales
9	0	0	1	0	1	Rhodospirillales
10	0	0	1	0	1	Rhodospirillales
11	1	0	0	0	1	Rhizobiales
12	1	0	0	0	1	Pseudomonadales

*For a complete description of all OTUs please refer to **Supplementary Table S3**.*

## Discussion

This study has revealed global scale patterns in diversity among hypolithic desert cyanobacteria, accompanied by a fairly cosmopolitan heterotrophic bacterial assemblage. Our sequencing depth exceeded that of previous studies on hypolithic systems and so we are confident of the community profiles generated. A rarefaction of our datasets to 12,000 filtered sequences per sample suggested that it was sufficient to inform >97% of the recoverable diversity in Taklimakan, Tibetan and Antarctic desert hypoliths, although for the more diverse Canadian Arctic sample this value was 88%. We therefore recommend 12,000 filtered reads as a minimum benchmark for future interrogation of the hypolithic system. It is also noteworthy that t-RFLP and pyrosequencing data were congruent in terms of identifying the 73 most abundant taxa for samples and so this validates the two-step approach using both these techniques for assessing desert communities where diversity and evenness are intrinsically low. Nonetheless with improved access to high throughput sequencing technology we recommend in-depth sequencing of all samples in any future study. The phylogeny of the Cyanobacteria is currently problematic due to the high level of phylogenetic plasticity within the phylum, apparent polyphyletic nature for some genera and potentially misidentified taxa based upon16S rRNA gene data in public databases. Nonetheless the topology for our phylogeny is congruent with generally accepted familial delineations (Komarek et al., 2014).

Hypolithic biofilms are distinct from the bacterial communities in surrounding soil (Pointing et al., 2009; Stomeo et al., 2013), although evidence suggests hypolithic biofilms are recruited at least in part from surrounding soil communities (Makhalanyane et al., 2012; Wei et al., 2016). In this study we have shown that desert hypolithic communities comprised just five main bacterial phyla with an extended ‘tail’ of taxa comprising less than 1% each of the overall community. A shared community architecture at the phylum level was dominated by cyanobacterial photoautotrophs, plus lower abundance of heterotrophic Acidobacteria, Actinobacteria, Bacteroidetes, Proteobacteria was consistent among all samples although relative abundance varied. This supports the findings of earlier studies based upon t-RFLP and sequence data from clone libraries that indicated similar community structure in hypoliths from China (Pointing et al., 2007) and Antarctica (Wood et al., 2008; Pointing et al., 2009). We did not encounter the red Chloroflexi-dominated hypoliths that occur infrequently in the Atacama Desert (Lacap et al., 2011) and our study excluded hypoliths with extensive moss development in the adjacent soil that are encountered in some cold (Wong et al., 2010) and polar (Cowan et al., 2010) deserts since the objective was to evaluate only the hypolithic biofilms.

The Antarctic hypolithic communities shared least similarity with those from all other deserts worldwide. This mirrors a global study of soil bacterial metagenomes where Antarctica was also shown to support a distinct community with genes associated with carbon catabolism and stress response accounting for much of the observed difference (Fierer et al., 2012). Recent studies of cryptic Antarctic communities including hypoliths suggests similar functional drivers may account for observed diversity in these systems (Chan et al., 2013; Wei et al., 2015, 2016). This delineation may reflect strong selective forces in Antarctica where microbial communities develop at or near the cold arid limit for life (Pointing et al., 2015; Goordial et al., 2016). A further factor may also be the geographic isolation and dispersal limitations to the Antarctic continent (Bottos et al., 2013; Pointing et al., 2015). Among all other desert locations worldwide communities resolved into three groupings that supported only a weak clustering according to location or climate. This may reflect relatively less environmental stress (Warren-Rhodes et al., 2006; Pointing et al., 2007; Stomeo et al., 2013) or dispersal limitations (Pointing and Belnap, 2014; Pointing et al., 2015), although limitations in sampling depth must also be considered in interpreting these data.

The cyanobacteria can be assumed to function as the primary producers in this system and their secretion of hygroscopic extracellular polymeric substance (EPS) is known as a major adaptation to moisture stress. This confers advantage to the whole community thus they are a keystone species in hypolithic communities (Billi et al., 2000; Warren-Rhodes et al., 2007). The ecological role of the heterotrophic component is poorly understood and only general conclusions about stress tolerance and adaptation to low energy environments for these taxa can be made since the phyla encountered are common to desert soils worldwide (Chanal et al., 2006; Connon et al., 2007; Pointing et al., 2009; Lee et al., 2012). The producers and consumers in the hypolithic system have been assumed to assemble under different niche and neutral processes. A stochastic demography for cyanobacterial distribution was postulated to reflect dispersal limitation, whilst the more cosmopolitan heterotrophs displayed patterns related to resource limitation (Caruso et al., 2011). We cannot discount other microclimate effects but these were unmeasured, although the hypolithic microclimate has been shown to vary little compared to the surrounding macroclimate features (Warren-Rhodes et al., 2006, 2007).

We introduced the application of a botanical concept, the growing season, a metric that reflects the number of days per year when a suite of macroclimate variables allow photosynthesis to occur (Paulsen et al., 2014). The P/C showed significant negative correlation to the growing season and this may reflect reduced productivity per unit of photoautotrophic biomass in low growing season locations necessary to sustain the community, since hypolithic heterotrophs are assumed to gain, at least in part, their nutrition from photosynthetic hypolithic exudates (Chan et al., 2012). It is worth noting that the range of P/C values was considerably broader in some samples and so this may have skewed the observed trend somewhat, although previous studies have also noted that hypolithic communities can be highly variable (Pointing et al., 2009). The growing season metric is potentially useful to apply to other microbial systems where producers and consumers are present and encompasses multiple macroclimatic variables. Whilst the samples were collected at different times that may not have coincided with an active growth period, the persistence of hypolithic communities over extended time periods is well documented (Warren-Rhodes et al., 2006). In this study we have assumed this was not a major source of error although it may be interesting in future study to investigate whether intra-growing season changes do occur.

Further consideration of cyanobacterial diversity revealed additional insight that may suggest a physiological basis for the observed trend in P/C ratio. Among the Cyanobacteria the notable observation was that cyanobacterial OTUs, whilst diverse, were largely dominated by two genera, *Chroococcidiopsis* in the Taklimakan Desert, and *Phormidium* in Tibetan and Antarctic deserts. The trend was pronounced between these three samples but the Arctic sample yielded a more diverse cyanobacterial assemblage dominated by poorly defined taxa largely from the Synechococcaceae. This may reflect the maritime location of this sample (Devon Island, Arctic tundra) since the genus *Synechococcus* has been identified in other maritime-associated desert hypoliths (Wei et al., 2016). It is interesting to speculate that *Phormidium* replaces *Chroococcidiopsis* as the dominant cyanobacteria as non-maritime deserts become colder and more extreme, such that in Antarctica hypolithic cyanobacteria are almost exclusively *Phormidium* sp. with no detectable *Chroococcidiopsis*. We rationalized the fast growing *Phormidium* is particularly adapted to Antarctic deserts where long periods of inactivity due to freezing are punctuated with short periods of favorable growth conditions where rapid colonization is an advantage, thus displaying elements of an R-selected strategy (Pointing, 2016). Conversely the *Chroococcidiopsis* taxa bear more resemblance to K-selected taxa that grow slowly but are specialized at exploiting highly xeric niches in hot and cold deserts due to copious EPS production and cellular desiccation tolerance mechanisms (Pointing, 2016). The broad delineation of macroclimate for these locations can potentially confuse this issue because they are based on mean annual values rather than actual growing season and so microclimate variation and the level of growth under xeric stress is not considered.

Further potential patterns for cyanobacterial occurrence extended beyond the dominant *Chroococcidiopsis* and *Phormidium* taxa to several other cyanobacteria across all families. Several taxa were encountered only in the two less extreme deserts whilst others were endemic to either extreme Taklimakan or Antarctic desert only. This supports the notion of stochastic demography for desert cyanobacteria observed in earlier studies (Bahl et al., 2011; Caruso et al., 2011), and may reflect a range of drivers such as environmental variables, dispersal limitation and legacy factors (Cowan et al., 2011a; Bottos et al., 2013; Pointing et al., 2014, 2015). This finding must be tempered with the fact that greater sampling depth may yield additional insight that may or may not support this. If ubiquitous dispersal is assumed then selection becomes a key issue for these cyanobacteria, and therefore suggests that deserts in different climatic regions have intrinsic conservation value based upon their endemic cyanobacteria. In the absence of data on the physiology of these taxa there is also a concern that any shift may give rise to dominance of certain ecotypes that may alter ecosystem function. This has been observed and predicted for soil microbial communities and is temperature dependent (Garcia-Pichel et al., 2013). A picture therefore emerges of desert hypoliths that assemble under strong selective pressure rather than dispersal limitation, although dispersal events for these communities in deserts are likely stochastic in nature (Warren-Rhodes et al., 2007; Pointing and Belnap, 2014). This contrasts with recent studies suggesting microbial dispersal is far greater than previously envisaged in marine systems (Gonnella et al., 2016) and so a single explanation for drivers of microbial biogeography remains elusive. Other explanations may apply to some taxa, for example the occurrence of OTUs indicating the chlorophyll d cyanobacteria and chlorophyte plastids. The chlorophyll d cyanobacterial genus *Acaryochloris* was encountered in a single location only and this may indicate a relatively low-light microhabitat beneath quartz for these samples, as they have been recovered from low light habitats in marine and desert locations (Mohr et al., 2010; Behrendt et al., 2011). The chlorophyte taxa occurred in all but the Taklimakan Desert and likely reflect free-living as well as lichenised taxa (Chan et al., 2012; Pointing, 2016). This is congruent with observations that chlorophytes are generally absent from deserts with high maximum temperatures but are more common in polar deserts (Chan et al., 2012).

The *nifH* sequence library suggested a significant role for non-cyanobacterial diazotrophy by Alphaproteobacteria, and this has been inferred from 16S rRNA gene taxonomic data in earlier study of the polar environment (Pointing et al., 2009) as well as acetylene reduction assays (Cowan et al., 2011b). Hypoliths may therefore emerge as potentially significant sites for nitrogen input to extreme desert systems and on a global scale as part of cryptogamic covers (Elbert et al., 2012). This is despite evidence that some extreme deserts may receive sufficient combined nitrogen from atmospheric deposition and perchlorate formation (Friedmann and Kibler, 1980; Andrew Jackson et al., 2015). One surprising finding was that despite recovery of low levels of *Nostoc* and other diazotrophic cyanobacteria, the *nifH* library yielded known diazotrophic taxa only from within the Proteobacteria. The PCR primers used are known to amplify cyanobacterial *nifH* genes and so this requires further investigation to fully resolve the contributing taxa to nitrogen fixation.

## Conclusion

Hypolithic microbial communities display patterns in diversity that appear to be driven largely by cyanobacterial adaptation to macroclimate and growing season. It seems reasonable to assume that diversity is determined by a complex interaction with multiple abiotic stressors and this could be a fruitful avenue for further research. The study also suggests that desert microbial communities may be far more diverse and regionally distinct than previously envisaged, and further work particularly with a focus on other domains of microbial life, may yield further insight (Pointing et al., 2016). The study also identifies that hypolithic communities are important reservoirs of low-abundance bacterial diversity in extreme deserts and these vary between locations, thus identifying a conservation value for these important desert communities.

## Author Contributions

SP, KW-R, and CM conceived the study. DL-B, ML, SP, and KW-R conducted fieldwork. DL-B, KL, SA, SL, and CL conducted laboratory experiments. DL-B, KL, SA, LG, TM, CM, JP, AdR-M, KW-R, DH, and SP analyzed data. DL-B, KL, SA, LG, TM, JP, AdR-M, KW-R, DH, and SP wrote the manuscript. All authors commented on and agreed the final manuscript.

## Conflict of Interest Statement

The authors declare that the research was conducted in the absence of any commercial or financial relationships that could be construed as a potential conflict of interest.

## Footnotes

1 www.clontech.com

2 www.thermofisher.com

3 https://products.appliedbiosystems.com

4 www.beckmancoulter.com

5 www.bmglabtech.com

6 https://www.roche.co.nz/

## Supplementary Material

TABLE S1 **Locations and macroclimate data for study sites.** Precise location for each sample was mapped via geographic information system to a global climate map based on long-term mean annual temperature and rainfall (Peel and Finlayson, 2007).

TABLE S2 **List of OTUs delineated using 454 pyrosequencing of the 16S rRNA gene.** OTUs were assigned following pyrosequencing data processing workflow described in Lee et al. (2012). The sequences were clustered at 97% sequence similarity.

TABLE S3 **List of OTUs delineated using 454 pyrosequencing of the *nifH* gene.** Sequences were processed following pyrosequencing data workflow described in Lee et al. (2012). The sequences were clustered at 97% similarity.
